# Distribution of vascular plants north of Lake Baikal: a new, open access dataset

**DOI:** 10.3897/BDJ.9.e77409

**Published:** 2021-12-14

**Authors:** Denis V. Sandanov, Elena P. Brianskaia, Eduard A. Batotsyrenov

**Affiliations:** 1 Institute of General and Experimental Biology SB RAS, Ulan-Ude, Russia Institute of General and Experimental Biology SB RAS Ulan-Ude Russia; 2 Baikal Institute of Nature Management SB RAS, Ulan-Ude, Russia Baikal Institute of Nature Management SB RAS Ulan-Ude Russia

**Keywords:** floristic studies, species occurrences, printed maps, Baikal-Amur Mainline, Russia, Siberia

## Abstract

**Background:**

The area north of Lake Baikal has been poorly studied. Moreover, most of the studies conducted in this region were focused on mountain ridges or river valleys. This region includes a part of Baikal-Amur Mainline (BAM), a broad-gauge railway in the centre of Siberia, Russia. The railway is an alternative route of the Trans-Siberian Railway; BAM starts in southern Siberia (Taishet station of Irktusk Oblast), passes through the northern part of Lake Baikal and finishes in the Russian Far East (Sovetskaya Gavan station of Khabarovsky Krai). BAM has four connections with the Trans-Siberian Railway and is the centre of economic development for many regions of Russia. Maya Ivanova and Alexandr Chepurnov summarised the existing floristic information for this region in detailed species distribution maps which they published in the book “Flora of the western part of developing regions of Baikal-Amur Mainline (BAM)” (1983). After publishing this book, very few floristic studies have been performed in the study region. All available botanical information is still accumulated in a number of printed papers or books with limited circulation, which are not widely known to the international scientific community.

**New information:**

We have digitised the point distribution maps from the book of Ivanova and Chepurnov and georeferenced all occurrence and sampling localities. The resulting dataset includes 9972 occurrences for 770 vascular plant species and subspecies from the area north of Lake Baikal. Additionally, the dataset includes information on the distribution of 43 rare and endangered species with 366 occurrences. From our point of view, the dataset makes a contribution to the global biodiversity data mobilisation, providing plant species distribution data for such a remote mountainous area.

## Introduction

**Figure 1.**
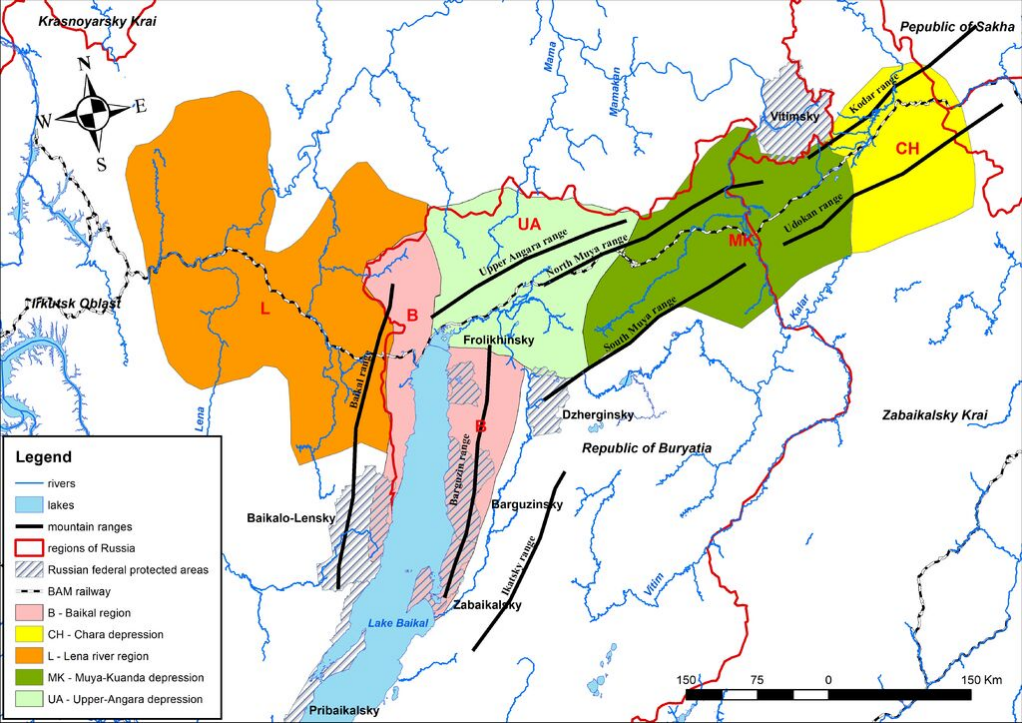
General map of the study area. The information on region topography, water bodies, floristic regions and protected areas are combined in one map. Floristic regions (according to Ivanova and Chepurnov 1983) are marked by colours.

Lake Baikal and its surrounding terrestrial ecosystems have recently undergone diverse climate change processes (Moore et al. 2009). The surface air temperature has warmed by 1.2°C during the last century; temperature increases have been observed in all seasons, but are greatest in winter and spring (Shimaraev et al. 2002). These changes are reflected in shifts in the phenology of vascular plants in the Barguzinsky Nature Reserve over the last 40 years with significant advances of spring events and delays of those associated with senescence in autumn (Rosbakh et al. 2021). The purpose of our work is to make available essential baseline data for analysing the manner in which the flora and vegetation around the northern end of Lake Baikal (Fig. 1) is responding to the global climate change. The northern part of this region includes a section of the Baikal-Amur Mainline (BAM), a broad-gauge railway line that goes through the centre of Siberia, which has led to the economic development along its route.

The mountains and river valleys around the northern part of Lake Baikal have been covered by a few botanical studies (Malyshev 1972, Tyulina 1976, Tyulina 1981, Ivanova and Chepurnov 1983). Ivanova and Chepurnov (1983) summarised previous floristic surveys and herbarium collections from the western part of BAM. They listed 1352 species and subspecies from 428 genera and 97 families occurring in the region. Species distribution maps for a larger area were published previously in “Alpine flora of Stanovoye Nagorye Upland” (Malyshev 1972) and "Flora of Central Siberia" (Malyshev and Peshkova 1979a, Malyshev and Peshkova 1979b).

Maps from these monographs have been critically analysed (Ivanova and Chepurnov 1983). In some cases, herbarium specimens were verified for clarification of species localities. New records of vascular species in the study area were added, based on specimens collected by N.S. Vodopyanova, M.M. Ivanova, Yu.N. Petrochenko, A.A. Chepurnov, M.G. Azovsky, V.V. Telyatiev and other researchers who worked at BAM (Table 1). These botanical studies were summarised in a book on the flora of the Baikal Siberian Region and its genesis (Malyshev and Peshkova 1984).

Recent botanical studies do not cover the whole area to the north of Lake Baikal. Even the publications that cover some parts of it (e.g. Upper Chara Depression (Garaschenko 1993); Kodar and Udokan Ranges (Shvetsova 2000); around Lake Baikal (Ivanova 2003); Vitimsky Nature Reserve (Czeczjotkin 1986, Czeczjotkin 1989, Chechyotkina 1993, Bardunov 2005, Chechyotkina 2010); and Baikalo-Lensky Nature Reserve (Stepantsova 2009, Stepantsova 2010, Stepantsova and Zheleznaya 2016) do not include distribution maps, being focused primarily on species lists or new species records in particular. Some recent publications do, however, provide geographical coordinates of species occurrences from satellite navigators.

The eastern part of BAM, the Chara floristic region, has been affected by large-scale human activities: copper mining in the Udokan Range, gold mining in the Olekma-Chara highland and proposals for extensions of BAM.

The development of portable satellite trackers has made incorporating georeference information into collection and observation records common. Our purpose for digitising the maps published in Ivanova & Chepurnov (1983) and freely sharing the resulting species occurrences is to provide the baseline data that will aid all those interested in the BAM's flora and in mapping its changes over time.

## General description

### Purpose

Digitising the vascular plant species distribution maps covering the western part of Baikal-Amur Mainline, which are published in Ivanova & Chepurnov (1983). This source contains crucial information on species distributions in the northern part of Lake Baikal, which is a less studied area of the Baikal Siberian Region. Other distribution maps currently available for this territory have a larger scale and many plant species are represented only by a few occurrences there.

## Project description

### Title

№121030900138-8 «Biota of terrestrial ecosystems of Baikal Region: composition, structure, eco-geographic patterns»

### Personnel

Denis Sandanov, Elena Brianskaia

### Study area description

Baikal Region, Russia

### Design description

The project is designed to benefit many different areas of study, such as: plant taxonomy, floristics, vegetation science, plant biology and population ecology, fauna and ecology of insects, ecology and geography of vertebrates.

### Funding

Russian Federal Budget

## Sampling methods

### Study extent

The study area is situated on the northern edges of three regions of Russia: Irkutsk Oblast, Republic of Buryatia and Zabaikalsky Krai. Some of the species occurrences at the north-western part of Lake Baikal, including Baikal Range, are now included in the Baikalo-Lensky Nature Reserve. The eastern part of the study area is legally protected in the Barguzinsky and Dzherginsky Nature Reserves, Zabaikalsky National Park and Frolikhinsky Sanctuary. The north-eastern part of Irkutsk Oblast includes the Vitimsky Nature Reserve (Fig. 1).

### Sampling description

**Figure 2.**
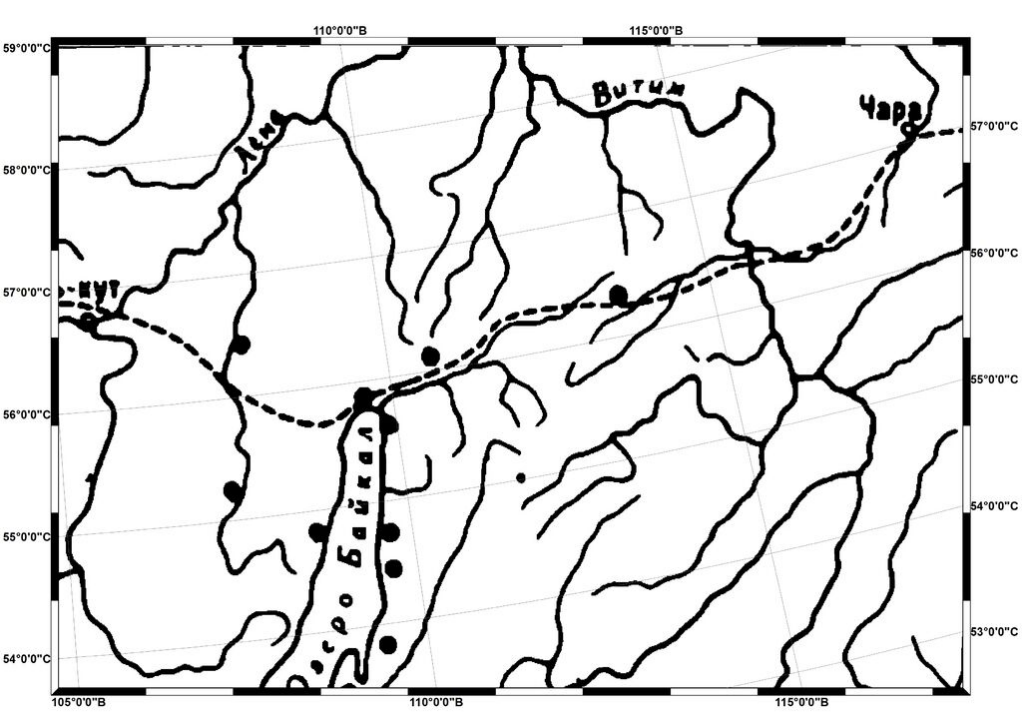
A georeferenced map (the distribution of *Matteucciastruthiopteris* (L.) Tod.) showing the study area.

In total, 770 maps were scanned from the book. Using the position of Lake Baikal and neighbouring rivers, we defined the projection of the maps (Fig. 2). We used a similar technique as employed for our previous dataset describing the distributions of endemic alpine species of northern Asia (Brianskaia et al. 2021). All the maps were adjusted to the same size and horizontal position in order to obtain standardised images of the maps. Digitalisation was performed in QGIS 3.10 software with the help of its georeferencing tools. The most accurate projected coordinate system was Asia North Albers Equal Area Conic. The water bodies shapefile was downloaded from the open source (https://vsegei.ru/ru/info/ggk_1000ns/) in scale 1:1 Mio. The river drainage shapefile fits very well with the original paper maps, but there were problems with the shape of Lake Baikal, especially in its northern part. In such cases, species distribution maps were georeferenced by snapping control points to the destination vector shapefile, which was the contour of Lake Baikal. We used control points (usually 5-8) to link maps to the destination shapefile, which resulted in the transformation of the maps according to the spatial projection of the destination features (WGS 1984). Subsequently, species distribution locations were digitised from each map. Coordinates of each location were calculated in the attribute table (Sandanov and Brianskaia 2021).

### Quality control

**Figure 3.**
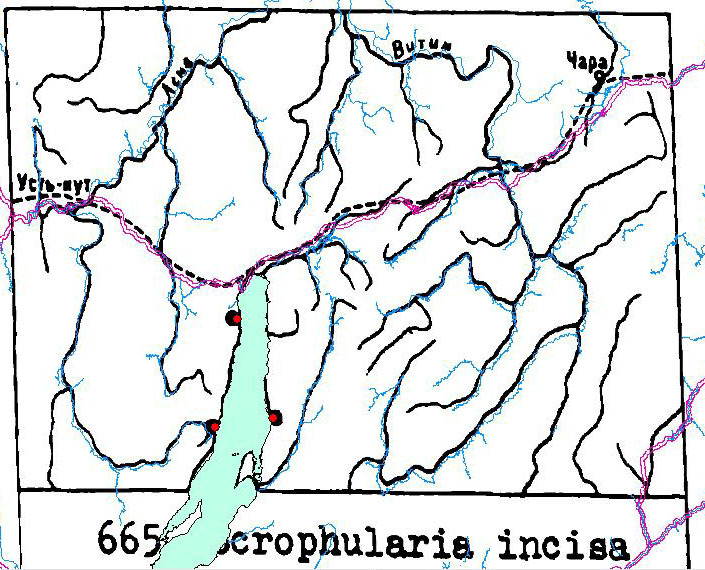
Georeferencing the distribution map of *Scrophulariaincisa* in QGIS 3.10. Overlay is the GIS shapefiles (denoted by colours), background is the original printed map (black and white).

We performed the final examination of the digitised species distribution maps in QGIS 3.10. For each species, we compared the output digitised occurrences with the original maps in order to check missing distribution records. The majority of occurrences (98%) matched consistently with the printed maps. Other 187 distribution records were manually adjusted for better matching with their habitats. These records mostly belong to the psammophytes occurring along the shoreline of Lake Baikal, especially at its northern part (Fig. 3). The diameter of points denoting the species occurrences is equal to 16 km. In this process, digitised localities of the psammophyte plants were moved closer to the shoreline. Taking this procedure into account, we estimate the coordinate uncertainty as 20 km for all the species in this study, taken as a matter of precaution.

## Geographic coverage

### Description

The study area includes the western part of BAM from Ust-Kut Town in the west and the Chara Depression in the east. It is a mountainous region involving several ranges of Stanovoy Highlands (Upper Angara, North Muya, South Muya, Kodar, Udokan), Baikal and Barguzin Ranges (Fig. 1). The main river of the study area is the Lena River. One of its southern tributaries is the Vitim River, which flows to the Lena from the north-east of Lake Baikal. The Vitim has tributaries draining the area, the Muya, Mamakan and Mama tributaries from the west and the Kalar and Kalakan tributaries from the east. The two major rivers on the west side of the region originate in the western part of the Stanovoy Highlands: the Upper Angara River that flows into the northern end of Lake Baikal and the Chaya River that flows into the Lena River.

The territory is divided into several floristic regions (Ivanova and Chepurnov 1983) (Fig. 1):

1. Lena River Region. Basins of Rivers Lena and Kirenga within Ust-Kutskii and Kazachinsko-Lenskii Districts of Irkutsk Oblast.

2. Baikal Region. This includes the north-western and north-eastern parts of Lake Baikal and is surrounded by the Baikal and Barguzin Ranges. It belongs to Severobaikalskii District of the Republic of Buryatia and partially to Kazachinsko-Lenskii District of Irkutsk Oblast.

3. Upper Angara Depression with Upper Angara Range. It is included in Severobaikalskii District of the Republic of Buryatia.

4. Muya-Kuanda Depression, which is bordered by the North Muya and South Muya Ranges. Most of this region is situated in Severobaikalskii and Bauntovskii Districts of the Republic of Buryatia, but the lowlands bordered on their right by the Vitim River belong to Kalarskii District of Zabaikalsky Krai.

5. Chara Depression with the Kodar, Udokan and Kalarsky Ranges. This is the eastern part of the Stanovoy Highlands. It does not include the Kuanda River, which is considered part of the Muya-Kuanda Depression floristic region. This region is located at the north of Kalarskii District of Zabaikalsky Krai.

**Figure 4.**
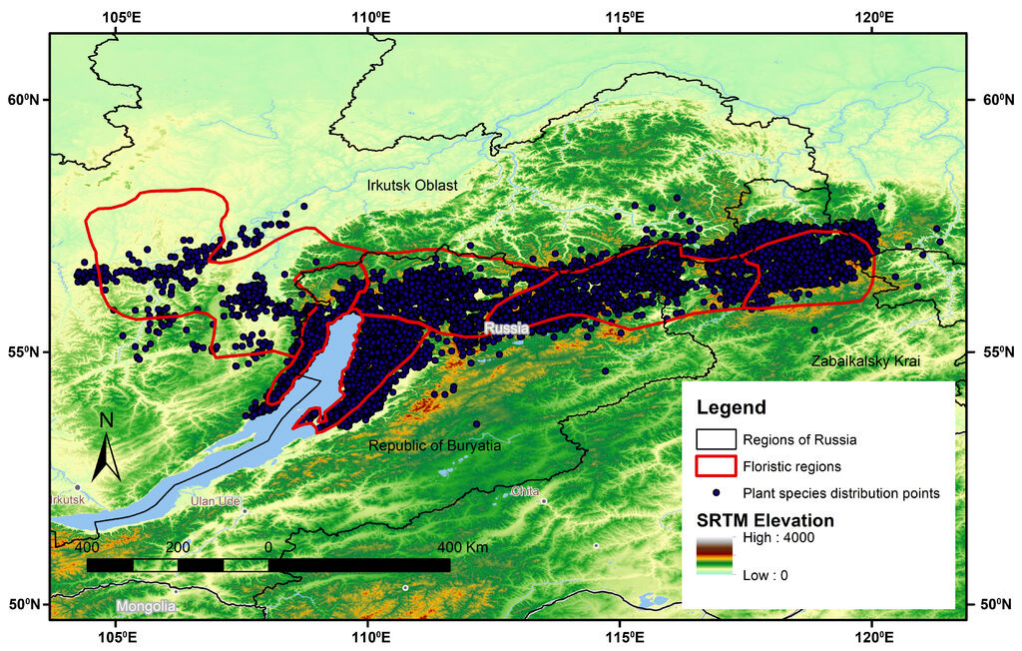
Location of vascular plants occurrences within the study area.

We mapped all localities recorded in the original printed maps. Most of these localities were included within the study area, but a few lay outside the digitised floristic regions (Fig. 4) due to the presence of general distribution data in the original maps.

### Coordinates

53.48 and 58.05 Latitude; 121.29 and 104.23 Longitude.

## Taxonomic coverage

### Description

The dataset includes 770 species and subspecies of vascular plants with 9972 occurrences from 81 families and 266 genera. The whole list of the flora of this region includes 1352 species and subspecies. Therefore, the dataset contains more than a half of the flora (57%) because the distribution maps were provided for the most common species only. In reporting the data, we retained the family attributions used in the source to facilitate comparisons. The top 10 families include 58.9% of the taxa and 56.9% of the occurrences (Table 2). In the original floristic analysis, *Scrophulariaceae* appeared in the top 10 families (Ivanova and Chepurnov 1983), but it is replaced by *Apiaceae* in our dataset. *Scrophulariaceae* in the current circumscription is represented in the dataset by only one species (Sandanov and Brianskaia 2021), *Scrophulariaincisa* (Fig. 3). Comparisons of percentages for all other families reveal further similarities between the complete floristic checklist and the species included in the dataset (Table 2). The comparisons testify that the dataset is representative for some part of the flora including families with high numbers of species. The digitised data can also be used for studies of the distribution patterns of key vascular plant species in the study region.

Our comparisons revealed that the list of top 10 genera was the same in the book and the dataset (Table 3). *Carex* and *Salix* are the leading genera in both lists. Standing next in the floristic list, *Potentilla* and *Artemisia* do not have distribution maps for the widely distributed species and that is why their position within the dataset is not so high. Other genera have similar positions as in the whole floristic checklist of the region.

The dataset contains information on the distribution of vascular plants species which are included in regional Red Data Books of the Baikal Siberian Region (Pronin 2013, Polyakov 2017, Trofimova 2020) (Table 4). These data are complementary to the recently-published dataset with occurrences of rare and endangered species of the Transbaikalia (Sandanov et al. 2021) and will be helpful in planning and implementing future conservation activities.

## Temporal coverage

### Notes

Dates of the specimen records used to prepare the printed maps ranged from 1912 to 1979 (Table 1).

## Usage licence

### Usage licence

Creative Commons Public Domain Waiver (CC-Zero)

### IP rights notes

This work is licensed under a Creative Commons Attribution (CC-BY) 4.0 Licence.

## Data resources

### Data package title

Occurrences of vascular plants in the western part of Baikal-Amur Mainline.

### Resource link

https://www.gbif.org/dataset/1e7b25d0-ec44-4fa8-8338-6e38e7a11214

### Alternative identifiers

1e7b25d0-ec44-4fa8-8338-6e38e7a11214; http://gbif.ru:8080/ipt/resource?r=vasc_plants_north_baikal

### Number of data sets

1

### Data set 1.

#### Data set name

Occurrences of vascular plants in the western part of Baikal-Amur Mainline.

#### Data format

Darwin Core Archive format.

#### Number of columns

29

#### Character set

UTF-8

#### Download URL

https://doi.org/10.15468/a8c783

#### Data format version

1.15

#### Description

The northern part of Lake Baikal has been sparsely covered by botanical studies which were usually concentrated on mountain ridges or river valleys. The floristic information for this region with point distribution maps of vascular plant species is summarised in the book by M.M. Ivanova and A.A. Chepurnov “Flora of the western part of developing regions of Baikal-Amur Mainline (BAM)” (Ivanova and Chepurnov 1983). All available maps from this book have been digitised and occurrences of vascular plants were organised in a dedicated dataset. The dataset includes 9972 occurrences for 770 vascular plant species and subspecies occurring around the northern part of Lake Baikal (the western part of Baikal-Amur Mainline), which is a hard-to-access mountainous region.

**Data set 1.** 

Column label	Column description
occurrenceID	An identifier for the record, unique within this dataset. An abbreviation in the identifier' number (IVBAM).
basisOfRecord	The specific nature of the data record in standard label of the Darwin Core classes: HumanObservation.
scientificName	The full scientific name of the species as recorded in the book by M.M. Ivanova and A.A. Chepurnov (1983) “Flora of the western part of developing regions of Baikal-Amur Mainline (BAM)”.
genus	The full scientific name of the genus in which the taxon is classified.
specificEpithet	The name of the species epithet as recorded in the book by M.M. Ivanova and A.A. Chepurnov (1983) “Flora of the western part of developing regions of Baikal-Amur Mainline (BAM)”.
infraspecificEpithet	The name of the lowest or terminal infraspecific epithet as recorded in the book by M.M. Ivanova and A.A. Chepurnov (1983) “Flora of the western part of developing regions of Baikal-Amur Mainline (BAM)”.
taxonRank	The taxonomic rank of the most specific name in the scientificName.
acceptedNameUsage	The full name, with authorship and date information, if known, of accepted taxon.
kingdom	The full scientific name of the kingdom in which the taxon is classified.
phylum	The full scientific name of the phylum or division in which the taxon is classified
class	The full scientific name of the class in which the taxon is classified.
order	The full scientific name of the order in which the taxon is classified.
family	The full scientific name of the family in which the taxon is classified.
decimalLatitude	The geographic latitude (in decimal degrees, using the spatial reference system given in geodeticDatum) of the geographic centre of a Location
decimalLongitude	The geographic longitude (in decimal degrees, using the spatial reference system given in geodeticDatum) of the geographic centre of a Location.
georeferencedBy	A list of persons who determined the georeference (spatial representation) for the Location.
geodeticDatum	The ellipsoid, geodetic datum or spatial reference system (SRS) upon which the geographic coordinates given in decimalLatitude and decimalLongitude are based.
eventDate	The date-time or interval during which an Event occurred. This is the publication date of the book by M.M. Ivanova and A.A. Chepurnov (1983) “Flora of the western part of developing regions of Baikal-Amur Mainline (BAM)”.
coordinateUncertaintyInMetres	The horizontal distance (in metres) from the given decimalLatitude and decimalLongitude describing the smallest circle containing the whole of the Location.
verbatimCoordinateSystem	The coordinate format for the verbatimLatitude and verbatimLongitude or the verbatimCoordinates of the Location.
higherGeography	A list of geographic names less specific than the information captured in the locality term.
continent	The name of the continent in which the Location occurs
country	The name of the country or major administrative unit in which the Location occurs.
countryCode	The standard code for the country in which the Location occurs.
type	The nature or genre of the resource.
language	A language of the resource.
licence	A legal document giving official permission to do something with the resource.
associatedReferences	A list (concatenated and separated) of identifiers (publication, bibliographic reference, global unique identifier, URI) of literature associated with the Occurrence.
taxonRemarks	Comments or notes about the taxon or name. Usually contains notes about definition of the taxon "sensu lato" or "sensu stricto" as recorded in the book by M.M. Ivanova and A.A. Chepurnov (1983) “Flora of the western part of developing regions of Baikal-Amur Mainline (BAM)”.

## Figures and Tables

**Table 1.** History of botanical studies in the northern part of Lake Baikal (from 1912 till 1979).

Territory	Botanists	Years of study
Ust-Kutskii District	Nomokonov L.I., Reshikov M.A. Popov M.G. and co-authors	1950 1951
Kazachinsko-Lenskii District	Alexandrov P. Belov A.V., Garashenko A.V. Azovsky M.G.	1912 1967 1979
Baikal Range and north-western part of Lake Baikal	Popov M.G., Malyshev L.I. Tyulina L.N., Ivanova M.M. Molozhnikov V.N., Granina G.T. Petrochenko Yu.N. Malyshev L.I. Makryi T.V. Telyatiev V.V.	1955 1958 1966 1966-1967 1967 1974 1979
Barguzin Range and north-eastern part of Lake Baikal	Tyulina L.N. Popov M.G. and coauthors Petrochenko Yu.N. Ivanova M.M. Malyshev L.I.	1939-1961 1954 1963, 1966 1965 1966-1968
Upper Angara Depression	Sukachev V.N., Poplavskaya G.I., Shipchinskii N.V. Malyshev L.I. Petrochenko Yu.N. Molozhnikov V.N. Ivanova M.M. Azovsky M.G.	1912 1955 1963, 1966 1975 1976 1977
Upper Angara Range	Petrochenko Yu.N. Malyshev L.I. Azovsky M.G.	1963, 1966 1966 1977, 1979
Muya-Kuanda Depression	Malyshev L.I., Petrochenko Yu.N. Ivanova M.M. Petrochenko Yu.N. Chepurnov A.A. Azovsky M.G.	1965 1967 1976 1977 1978
North Muya Range	Petrochenko Yu.N. Malyshev L.I., Petrochenko Yu.N. Ivanova M.M. Malyshev L.I., Petrochenko Yu.N.	1963 1965 1967 1968
South Muya Range	Malyshev L.I., Petrochenko Yu.N. Ivanova M.M., Andrulaitis S.Yu. Petrochenko Yu.N.	1966 1966-1967 1968
Chara Depression	Mikheev V.S. Garashenko A.V. Malyshev L.I., Petrochenko Yu.N. Vodopyanova N.S. Chepurnov A.A.	1963-1964 1964, 1975-1976 1964 1967 1978
Kodar Range	Malyshev L.I., Petrochenko Yu.N. Vodopyanova N.S. Chepurnov A.A.	1964 1967 1978
Udokan Range	Vodopyanova N.S. Ivanova M.M., Bardunov L.V. Chepurnov A.A.	1964-1967 1969 1978

**Table 2.** Taxonomic distribution of vascular plants in the northern part of Lake Baikal. Families are listed in descending order of the number of species and subspecies.

No.	Family	No. of species and subspecies	No. of records	Percent of species and subspecies (dataset)	Percent of species and subspecies (Ivanova and Chepurnov 1983)
1	Poaceae	95	1104	12.3	9.5
2	Cyperaceae	84	1075	10.9	9.3
3	Asteraceae	64	661	8.3	10.6
4	Caryophyllaceae	38	495	4.9	4.2
5	Ranunculaceae	33	411	4.3	5.2
6	Rosaceae	32	414	4.2	5.5
7	Salicaceae	31	728	4	3
8	Brassicaceae	29	272	3.8	4.1
9	Fabaceae	27	269	3.5	4.6
10	Apiaceae	20	249	2.6	2.5
11	Juncaceae	17	256	2.2	1.6
12	Orchidaceae	17	116	2.2	1.7
13	Orobanchaceae	17	216	2.2	0.2
14	Saxifragaceae	17	424	2.2	2.4
15	Ericaceae	16	299	2.1	1.3
16	Boraginaceae	14	125	1.8	1.4
17	Polygonaceae	12	210	1.6	1.9
18	Lamiaceae	11	98	1.4	2.2
19	Potamogetonaceae	11	76	1.4	1.1
20	Violaceae	9	125	1.2	1.1
21	Lycopodiaceae	8	190	1	0.6
22	Betulaceae	7	183	0.9	1.2
23	Gentianaceae	7	102	0.9	1.5
24	Caprifoliaceae	6	116	0.8	0.4
25	Cystopteridaceae	6	120	0.8	0.4
26	Papaveraceae	6	45	0.8	0.9
27	Plantaginaceae	6	41	0.8	0.3
28	Typhaceae	6	45	0.8	0.1
29	Amaryllidaceae	5	39	0.7	0.4
30	Campanulaceae	5	117	0.7	0.4
31	Crassulaceae	5	76	0.7	0.6
32	Geraniaceae	5	52	0.7	0.7
33	Liliaceae	5	54	0.7	2.1
34	Polemoniaceae	5	77	0.7	0.4
35	Primulaceae	5	49	0.7	1
36	Dryopteridaceae	4	58	0.5	0.4
37	Grossulariaceae	4	73	0.5	0.3
38	Lentibulariaceae	4	34	0.5	0.5
39	Araceae	3	12	0.4	0.1
40	Athyriaceae	3	52	0.4	0.7
41	Iridaceae	3	14	0.4	0.2
42	Melanthiaceae	3	51	0.4	0.2
43	Montiaceae	3	29	0.4	0.2
44	Nymphaeaceae	3	20	0.4	0.3
45	Onagraceae	3	54	0.4	0.4
46	Pinaceae	3	70	0.4	0.5
47	Rubiaceae	3	17	0.4	0.6
48	Selaginellaceae	3	45	0.4	0.3
49	Tofieldiaceae	3	51	0.4	0.2
50	Adoxaceae	2	21	0.3	0.1
51	Alismataceae	2	20	0.3	0.2
52	Amaranthaceae	2	10	0.3	0.1
53	Aspleniaceae	2	13	0.3	0.2
54	Cupressaceae	2	39	0.3	0.1
55	Juncaginaceae	2	18	0.3	0.2
56	Ophioglossaceae	2	19	0.3	0.2
57	Polygalaceae	2	13	0.3	0.1
58	Pteridaceae	2	39	0.3	0.3
59	Thelypteridaceae	2	18	0.3	0.1
60	Woodsiaceae	2	36	0.3	0.1
61	Acoraceae	1	4	0.1	0.1
62	Asparagaceae	1	28	0.1	0.1
63	Ceratophyllaceae	1	6	0.1	0.1
64	Dennstaedtiaceae	1	6	0.1	0.1
65	Diapensiaceae	1	29	0.1	0.1
66	Droseraceae	1	6	0.1	0.1
67	Ephedraceae	1	12	0.1	0.1
68	Equisetaceae	1	29	0.1	0.6
69	Euphorbiaceae	1	32	0.1	0.1
70	Haloragaceae	1	6	0.1	0.1
71	Hydrocharitaceae	1	5	0.1	0.1
72	Isoetaceae	1	6	0.1	0.1
73	Linaceae	1	6	0.1	0.1
74	Lythraceae	1	2	0.1	0.1
75	Menyanthaceae	1	5	0.1	0.1
76	Onocleaceae	1	10	0.1	0.1
77	Polypodiaceae	1	11	0.1	0.1
78	Scheuchzeriaceae	1	3	0.1	0.1
79	Scrophulariaceae	1	3	0.1	3
80	Tamaricaceae	1	4	0.1	0.1
81	Urticaceae	1	4	0.1	0.4
Total		770	9972	-	-

**Table 3.** Top 10 genera within the study area. Genera are listed in descending order of the number of species.

№	Genera	No. of species and subspecies (dataset)	No. of records (dataset)	Percent of species and subspecies (dataset)	Percent of species and subspecies (Ivanova and Chepurnov 1983)
1	*Carex*	66	852	24.8	23.8
2	*Salix*	30	708	11.3	8.6
3	*Poa*	16	251	5.6	4.9
4	*Saxifraga*	14	382	5.3	4.7
5	*Potentilla*	13	175	4.9	7.0
6	*Pedicularis*	10	140	3.8	4.2
7	*Astragalus*	9	127	3.4	4.0
8	*Oxytropis*	9	55	3.4	4.2
9	*Artemisia*	7	93	2.6	6.3
10	*Polygonum*	6	114	2.3	4.2

**Table 4.** The list of vascular plant species included in regional Red Data Books of the Baikal Siberian Region.

Species	Number of records	Region, where the species is considered rare and endangered
*Arctousalpina*	8	Zabaikalsky Krai
*Atrageneochotensis*	9	Zabaikalsky Krai
*Borodiniamacrophylla*	9	Irkutsk Oblast, Republic of Buryatia, Zabaikalsky Krai
*Calypso bulbosa*	4	Irkutsk Oblast, Republic of Buryatia, Zabaikalsky Krai
*Caraganajubata*	6	Republic of Buryatia, Zabaikalsky Krai
*Carexmalyshchevii*	6	Zabaikalsky Krai
*Carexsabulosa*	3	Zabaikalsky Krai
*Cotoneasterneo-popovii*	4	Irkutsk Oblast, Republic of Buryatia
*Cotoneastertjuliniae*	5	Republic of Buryatia
*Craniospermumsubvillosum*	9	Irkutsk Oblast, Republic of Buryatia
*Cypripediumcalceolus*	5	Irkutsk Oblast, Republic of Buryatia, Zabaikalsky Krai
*Cypripediumguttatum*	11	Zabaikalsky Krai
*Cypripediummacranthon*	4	Irkutsk Oblast, Republic of Buryatia, Zabaikalsky Krai
*Deschampsiaturczaninowii*	8	Irkutsk Oblast, Republic of Buryatia
*Epipactishelleborine*	2	Irkutsk Oblast, Republic of Buryatia
*Epipogiumaphyllum*	6	Irkutsk Oblast, Republic of Buryatia, Zabaikalsky Krai
*Gastrolychnispopovii*	4	Republic of Buryatia
*Isoetessetacea*	6	Republic of Buryatia, Zabaikalsky Krai
*Liliumpilosiusculum*	8	Zabaikalsky Krai
*Liliumpensylvanicum*	7	Zabaikalsky Krai
*Liliumpumilum*	3	Zabaikalsky Krai
*Listeracordata*	3	Republic of Buryatia
*Lycopodiumjuniperoideum*	6	Irkutsk Oblast, Republic of Buryatia, Zabaikalsky Krai
*Mertensiaserrulata*	6	Republic of Buryatia
*Neottiacamtschatea*	3	Irkutsk Oblast, Republic of Buryatia
*Neottianthecucullata*	2	Republic of Buryatia
*Nymphaeacandida*	2	Irkutsk Oblast, Republic of Buryatia
*Nymphaeatetragona*	12	Irkutsk Oblast, Republic of Buryatia, Zabaikalsky Krai
*Oxytropiskodarensis*	4	Irkutsk Oblast, Zabaikalsky Krai
*Phlojodicarpusvillosus*	10	Zabaikalsky Krai
*Phyllodocecoerulea*	2	Zabaikalsky Krai
*Potentillaadenotricha*	5	Zabaikalsky Krai
*Pulsatillaajanensis*	5	Republic of Buryatia, Zabaikalsky Krai
*Rhodiolaquadrifida*	28	Zabaikalsky Krai
*Rhodiolarosea*	35	Irkutsk Oblast, Republic of Buryatia, Zabaikalsky Krai
*Rhododendronadamsii*	23	Irkutsk Oblast, Republic of Buryatia, Zabaikalsky Krai
*Rhododendronaureum*	45	Zabaikalsky Krai
*Rhododendronredowskianum*	35	Zabaikalsky Krai
*Rhynchosporaalba*	3	Irkutsk Oblast, Republic of Buryatia, Zabaikalsky Krai
*Ribesdikuscha*	4	Irkutsk Oblast, Republic of Buryatia
*Tillaeaaquatica*	2	Irkutsk Oblast, Republic of Buryatia
*Trapanatans*	2	Zabaikalsky Krai
*Zannichelliarepens*	2	Irkutsk Oblast, Republic of Buryatia
Total (43 species)	366	-
